# Hyperthermic Intraperitoneal Chemotherapy in Gastric Cancer: A Clinical Case Involving Long-Term Survival

**DOI:** 10.7759/cureus.45302

**Published:** 2023-09-15

**Authors:** Ana Duarte Mendes, Rodrigo Vicente, Manuel Fernandes, Michelle Silva

**Affiliations:** 1 Oncology, Hospital Professor Doutor Fernando Fonseca, Amadora, PRT; 2 General Surgery, Instituto Português de Oncologia do Porto, Porto, PRT

**Keywords:** long-term survival, case report, gastric cancer, peritoneal carcinomatosis, hyperthermic intraperitoneal chemotherapy

## Abstract

Peritoneal metastasis is the most common pattern of synchronous and metachronous dissemination in gastric cancer (GC) and is associated with a poor prognosis. Even though systemic chemotherapy is the standard of care, the optimal therapeutic approach to peritoneal disease in this setting is yet to be defined. We present a case of a 26-year-old female diagnosed with locally advanced GC who developed peritoneal carcinomatosis (PC). The patient underwent cytoreductive surgery (CRS) along with hyperthermic intraperitoneal chemotherapy (HIPEC) with complete remission. She remained disease-free after six years, presenting with peritoneal recurrence 70 months after the procedure. This report describes a rare case of long-term survival following a controversial therapeutic approach.

## Introduction

Gastric cancer (GC) is the sixth most common cancer worldwide and the fourth leading cause of death by cancer in both sexes [1]. In 2020, GC incidence surpassed one million new cases, with over two-thirds of them reported in Asia. In Europe, incidence rates are much lower, and there is also a geographical variation between Eastern and Northern European countries, with lower incidence reported in the latter [1]. The incidence has been declining due to improvements in hygiene and the successful eradication of Helicobacter pylori infection. Even though survival rates have improved due to earlier detection and better treatment options, GC remains a deadly disease, with approximately 760,000 deaths reported in 2020 [1,2].

Gastric cancers are common gastrointestinal malignancies that peak in prevalence in males over 50 years of age. Several environmental factors are often linked to GC, such as Helicobacter pylori infection, diet, smoking, and binge drinking [3]. There is a lack of consensus on the definition of early-onset GC, but one of the most accepted definitions pertains to those diagnosed at 50 years or younger. Approximately 10-30% of GC cases are defined as early-onset. Even though there is discordant evidence regarding sex predominance, the condition has been observed more frequently in females. It has been associated with particular clinical and pathological characteristics, such as a predominance of diffuse histology and infrequent association with intestinal metaplasia. In this setting, genetic factors may play a pivotal role [4,5]. The peritoneum and the liver are the most frequent sites for GC metastasis [6]. Peritoneal carcinomatosis (PC) is responsible for 35% of all synchronous metastases, and peritoneal recurrence after curative surgery occurs in up to 46% of patients. PC translates into poor survival rates, with a median survival of only four months [7].

PC remains a clinical problem for which therapeutic options remain scarce. The poor response to systemic chemotherapy has led to the development of regional therapies, namely cytoreductive surgery (CRS) and hyperthermic intraperitoneal chemotherapy (HIPEC) [7-10]. These combined modalities are the standard of care for peritoneal mesothelioma and pseudomyxoma peritonei and have also been used to treat peritoneal metastases from colorectal cancer in select patients [10]. Extensive multicenter studies in colorectal cancer patients have shown a median survival of 30-40 months [11]. The principles of this approach have been thoroughly described by Sugarbaker [12], which are as follows: (1) selecting patients whose disease is limited to the surface of the abdomen or pelvis and (2) performing CRS to maximally reduce disease burden by resecting involved viscera plus peritonectomy.

We present the clinical case of a young patient with GC who underwent CRS + HIPEC in 2016 and remained disease-free until June 2022.

## Case presentation

In March 2015, an otherwise healthy 26-year-old Caucasian woman was admitted to our emergency department with complaints of three months of fasting epigastric pain, anorexia, and weight loss (described as 5% of her weight). She had no history of smoking or alcohol abuse. Regarding her family medical history, two maternal relatives had a history of cancer: her grandmother had biliary tract cancer, and her aunt had gastric cancer. 

**Figure 1:**
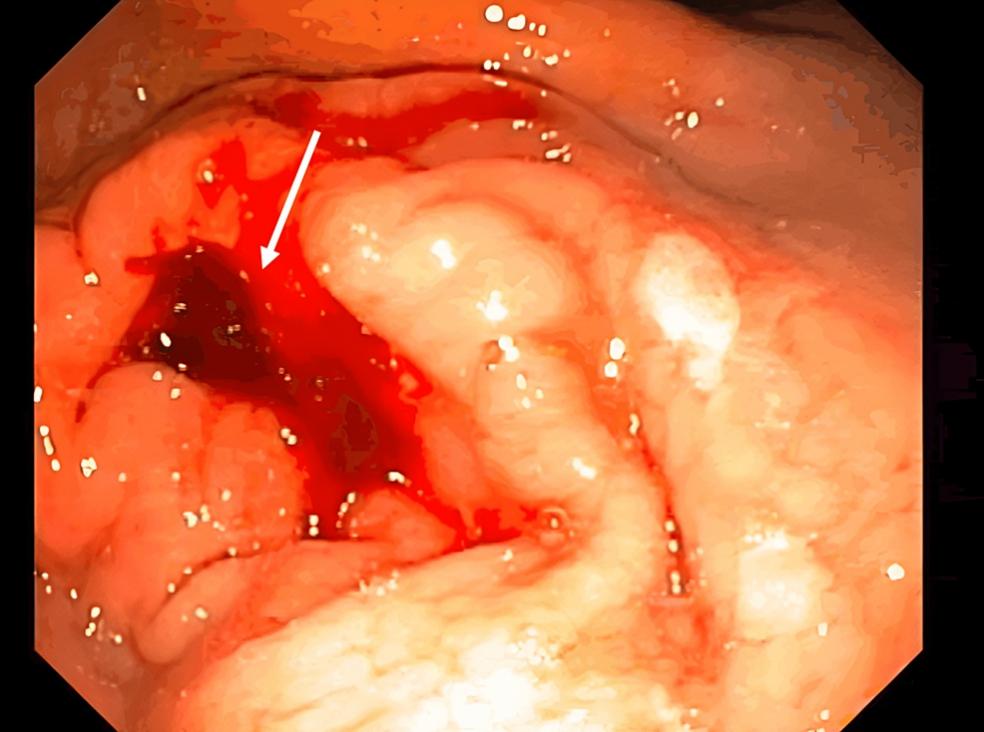
Upper endoscopy at diagnosis The initial upper endoscopy identified a 15 x 15 mm Forrest III gastric ulcer in the pyloric antrum (white arrow), with a pearly bottom and congestive edges

The initial diagnostic workup showed a low hemoglobin count (6.0 g/dL), and the upper endoscopy identified a 15 x 15 mm Forrest III gastric ulcer in the pyloric antrum (Figure 1).

**Figure 2:**
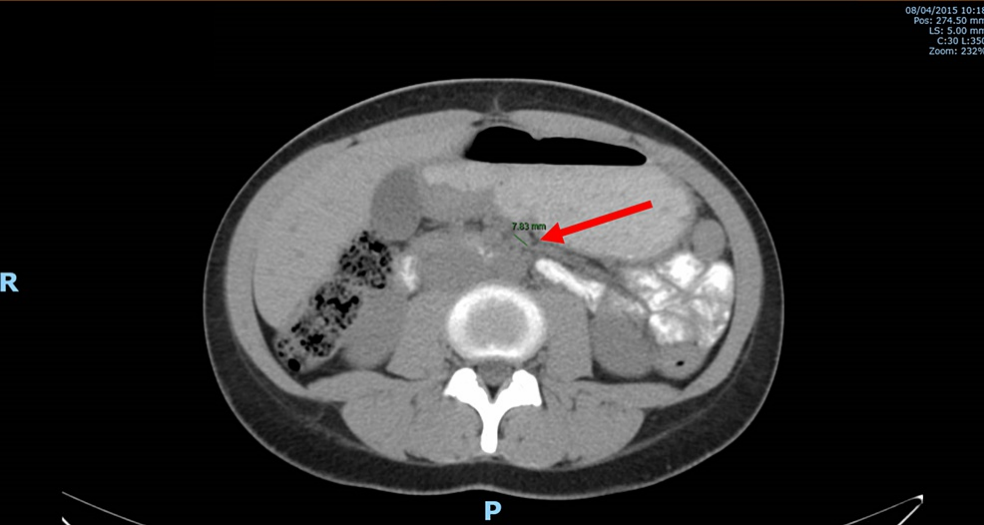
CT scan at diagnosis The initial CT scan showed no signs of metastatic disease, but small nodes were noted in the retrogastric adipose layer, the largest being 7.83 mm in diameter (red arrow) CT: computed tomography

**Figure 3:**
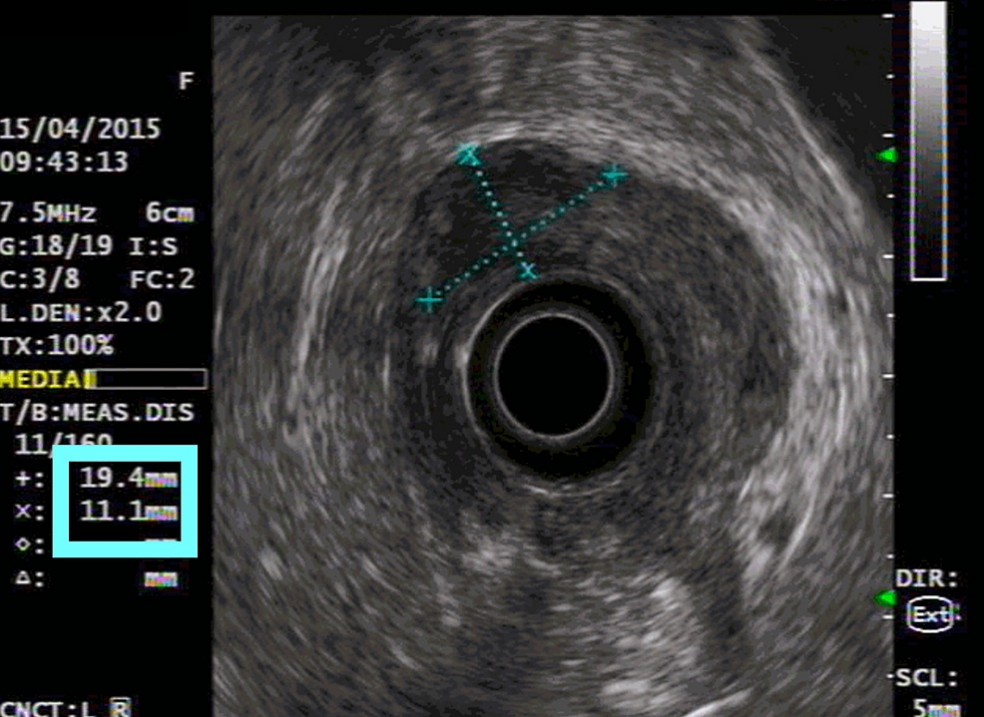
Endoscopic ultrasound at diagnosis We performed an endoscopic ultrasound (EUS) for the locoregional disease staging, with an estimated depth of invasion of 19.4 mm (blue box)

The biopsy revealed a poorly cohesive carcinoma (WHO Classification, 2010), with neither microsatellite instability nor HER overexpression. The endoscopic ultrasound (EUS) and CT scan established the diagnosis of locally advanced gastric cancer (TNM classification: T2 N+ M0, stage II/III) (Figures 2, 3). Staging laparoscopic and peritoneal cytology was negative for malignant cells. The patient was referred to a genetic counseling consultation to rule out hereditary diseases.

The multidisciplinary team proposed perioperative chemotherapy with epirubicin, cisplatin, and 5-fluorouracil (ECF), based on the MAGIC trial, which the patient accepted. She started chemotherapy in May 2015, which was well tolerated with no significant toxicities. In September 2015, the patient underwent total gastrectomy with D2 lymph node dissection. Histological examination confirmed a poorly cohesive carcinoma in the pyloric antrum extending to the serosa and regional lymph node involvement in three out of 47. The distal margin (duodenal) was also involved (pT4 pN2 R1). Given the morbidity of a duodenopancreatectomy to obtain an R0 margin and the high risk of disease dissemination due to its aggressive characteristics, the patient was proposed to undergo systemic therapy with subsequent imaging evaluation. Given that the pathological response to ECF was negligible, we decided to change the chemotherapy regimen. Modified docetaxel, cisplatin, and 5-fluorouracil (DCF) were chosen because several studies had shown a better response rate vs. ECF. She completed three cycles of DCF from November 2015 to January 2016, with moderate tolerance (grade 1 mucositis, grande 2 peripheral sensory neuropathy, and grade 2 neutropenia). In February 2016, the patient underwent a PET CT scan, which showed no foci of hypermetabolism.

Due to the high risk of peritoneal dissemination (pT4N2R1, diffuse histology) and given that the imaging evaluation by PET CT scan has reduced sensitivity in this histology, the patient was proposed for a new staging laparoscopy. In March 2016, she underwent surgery. Intraoperatively, voluminous ascites and two small foci (2 mm) of peritoneal metastasis were detected, which were removed. The peritoneal cancer index (PCI) was established as 2. A left oophorectomy was also performed due to a stony-hard mass in the left ovary. Histological examination confirmed the suspicion of PC and diagnosed a Krukenberg tumor of the left ovary. Considering that the patient presented exclusively with abdominal progression, it was proposed that she should undergo CRS + HIPEC at a dedicated center.

In August 2016, she underwent complete CRS (CC-0), which included a right oophorectomy and the excision of multiple peritoneal nodules, and HIPEC with mitomycin 35 mg/m^2^ for 90 minutes, which led to no significant complications. Considering that the patient responded poorly to the chemotherapy regimens previously, it was decided not to perform adjuvant therapy. She underwent a CT scan in December 2016, which confirmed the complete remission of the disease. 

Our patient remained disease-free until June 2022, when she presented at the emergency department with an episode of small bowel subocclusion due to extensive peritoneal recurrence. A peritoneal biopsy revealed a poorly cohesive carcinoma (WHO Classification, 2010) with no microsatellite instability, HER overexpression, or actionable mutation. She was started on systemic therapy in July 2022 with ramucirumab and irinotecan, with disease progression in June 2023. She is currently undergoing chemotherapy with paclitaxel and remains clinically stable.

## Discussion

In patients with PC, it is essential to stage the extent of the disease to select the proper treatment, and PCI is used for this purpose. It involves dividing the abdomen into nine quadrants plus four regions, including the small bowel. Each region is given a score based on the largest implant size (LS 0-3), where LS-0 means no lesion is seen, LS-1 stands for lesions up to 0.5 cm, LS-2 means up to 5 cm, and LS-3 indicates implants greater than 5 cm. Accurately detecting the extent of peritoneal cancer in GC patients remains a challenge. Despite no signs of cancer spread on preoperative imaging, many patients may still have it when diagnosed through laparoscopy. Additionally, patients may have positive peritoneal cytology even if laparoscopy indicates no visible disease. These findings underscore the inadequacy of our current diagnostic methods and reinforce the importance of precisely identifying cancer burden to prevent postoperative complications resulting from unsuccessful surgeries [12].

Considering the high incidence of PC in GC, these modalities have also been applied in three potential settings: as a prophylactic approach after a curative gastrectomy in high-risk patients (in an adjuvant-like manner), as a treatment option for patients with established PC, and as a palliative approach for patients with symptomatic PC [7]. For GC, this technique was first described by Fujimoto et al. [13] in 1988, performing CRS in 15 patients with advanced GC, nine of whom had PC. The PC resolved, and peritoneal lavage cytology in all nine patients became negative. A few other reports have emerged, mainly from Japan, and in a study in 1997, Fujimoto et al. [14] reported a significantly higher five-year survival in the CRS/HIPEC group (31% vs. 0%, p=0.001).

In Europe, a multicentric study involving 15 centers (France and Belgium) by Glehen et al. [9] reported a five-year survival of 13% and a median survival of 9.2 months. The inconsistencies in technique, drug use, temperature, and HIPEC duration compromise these results' reliability. Intraperitoneal chemotherapy (IPC) may also be effective as a targeted adjuvant treatment. A recent meta-analysis including 20 prospective randomized controlled trials involving 2,145 patients demonstrated that IPC improves survival and decreases the incidence of peritoneal failure or distant metastasis compared to surgery alone [15]. In the palliative setting, HIPEC has been successfully used to alleviate ascites caused by GC. Laparoscopic HIPEC has recently emerged as an effective solution for patients struggling with intractable and debilitating ascites due to GC. In a systematic review of five studies involving 76 patients, 37 of them having GC, laparoscopic HIPEC successfully controlled ascites in 95% of cases. The authors also reported a low incidence of minor complications, with no significant complications observed [16].

A novel technique that has sparked interest is utilizing high pressure within the peritoneum to deliver heated chemotherapy, referred to as pressurized intraperitoneal aerosol chemotherapy (PIPAC). The concept behind this approach is that the heightened pressure will enhance the absorption of chemotherapy drugs, while the aerosolization will offer more comprehensive coverage of the affected region, resulting in a superior anti-tumor outcome. PC carries a poor prognosis in GC. To date, classical chemotherapy remains the gold standard. However, we are aware that the therapeutic response to systemic chemotherapy falls far short of what is desired due to several mechanisms. The "plasma-peritoneal barrier" and the poor intraperitoneal blood supply possibly contribute to the poor response to chemotherapy [17]. Effective regional treatment is essential to guide appropriate further therapy in advanced GC patients.

The rationale for using hyperthermia relies on three mechanisms: the direct cytotoxic effect of heat on cancer cells, the synergistic effect with specific chemotherapy agents, and finally, the improvement of penetration of chemotherapy, which results in increased sensitivity of tumor cells to chemotherapy. The primary objective of CRS is to eliminate all visible peritoneal lesions, leaving HIPEC to only target free-floating cancer cells and micro-metastases on the peritoneum. Any nodule more prominent than 1-2 mm must be surgically removed during CRS since the penetration of drugs in HIPEC is limited to that depth. The use of agents such as mitomycin C, cisplatin, or doxorubicin, which demonstrate increased efficacy with heat, is paramount. The heat also helps to improve drug uptake by impairing DNA repair mechanisms in tumor cells and inducing apoptosis through the denaturation of proteins. The blood-peritoneal barrier allows large doses of cytotoxic drugs to be administered without significant systemic side effects [7,9].

An optimal CRS is the bedrock for this regional modality's success, allowing the removal of the whole macroscopic disease. The benefit of HIPEC is still a matter of controversy, mainly due to inconsistent protocols and lack of randomized trials [14]. As stated above, our patient was diagnosed with locally advanced gastric cancer, with poor response to perioperative chemotherapy and exclusive abdominal progression. She underwent thorough cytoreduction and HIPEC and remained disease-free and clinically well after almost six years. 

It is worth noting that there have been a handful of reports of long-term survival, with Brandl et al. [18] addressing relevant questions as to which patient characteristics may be associated with a better outcome. The completeness of cytoreduction (CC-0) and a low PCI (<6) are relevant to the outcome [18,19]. For patients with a CC-0, the five-year overall survival ranges from 13% to 23% [18]. Our patient had a low PCI and complete cytoreduction, which may have been critical to her long-term outcome. In the context of HIPEC, there is another question that needs to be raised regarding our patient: what is the best post-recurrence approach? There is limited information available about the recurrence patterns and treatment options for GC following HIPEC. However, patients could benefit from additional treatment, such as systemic therapy, surgery on non-peritoneal metastases (such as liver and lung resections), and secondary CRS.

A new surgical intervention was unfeasible since it was an extensive peritoneal recurrence. Therefore, we proposed systemic therapy. The patient had shown reasonable tolerability to DCF but still had residual neurotoxicity, prompting us to postpone the reintroduction of taxanes. We suggested systemic therapy with ramucirumab and irinotecan, given its promising anticancer activity in subsequent lines [20].

## Conclusions

Standardized studies are crucial for providing compelling evidence about the effectiveness of HIPEC with CRS. The current evidence, which is mainly drawn from small retrospective or prospective studies, is insufficient and calls for more extensive research. Our report describes a rare case of long-term disease-free survival with this controversial therapeutic approach. Further studies are warranted on this topic.
